# How traditional Chinese exercise (Daoyin) can help COVID-19 patients relieve psychological symptoms: a systematic review and meta-analysis

**DOI:** 10.3389/fpsyt.2024.1422229

**Published:** 2024-08-13

**Authors:** Naifan Duan, Feng Chen, Yalan Li, Linqiong Zhou, Xing Zhang, Guihua Xu, Wei Zhang

**Affiliations:** ^1^ Department of Pulmonary Diseases, Shuguang Hospital Affiliated to Shanghai University of Traditional Chinese Medicine, Shanghai, China; ^2^ Division of Pulmonary, Critical Care, Allergy, and Sleep, Department of Medicine, University of California, San Francisco, San Francisco, CA, United States

**Keywords:** COVID-19, Daoyin, traditional Chinese medicine, SAS, SDS, meta-analysis

## Abstract

**Objective:**

The mental health issues of individuals with coronavirus disease 2019 (COVID-19) are currently widespread. Traditional Chinese exercise (Daoyin) plays an important role in relieving patients’ psychological problems. This study aims to assess the efficacy of Daoyin in mitigating mental health issues among individuals diagnosed with COVID-19.

**Methods:**

PubMed, the Cochrane library, Embase, CNKI, Wanfang, VIP database, and SinoMed were searched from their inception to October 2023. Two researchers independently selected the eligible studies. The analysis and presentation of the findings were conducted using Review Manager 5.2 software. The data were analyzed using mean difference (MD), inverse variance, and 95% confidence intervals (CIs).

**Results:**

A total of 12 studies (*N* = 1291) were included in this study. The results showed that Daoyin can significantly reduce the scores of the Self-Rating Anxiety Scale (SAS: MD = −13.03, 95% CI −19.56 to −6.49, *P*<.49,yca Self-Rating Depression Scale (SDS: MD = −11.13, 95% CI −14.56 to −7.71, *P*<.71,sion Pittsburgh Sleep Quality Index (PSQI: MD = −2.00, 95% CI −5.43 to 1.43, *P* = 0.25), Hamilton Anxiety Scale (HAMA: MD = −2.42, 95% CI −5.25 to 0.41, *P* = 0.09), and Hamilton Depression Scale (HAMD: MD = −11.17, 95% CI −25.5 to 3.15, *P* = 0.13).

**Conclusion:**

In COVID-19 patients, Daoyin can alleviate feelings of anxiety and depression, as well as improve sleep quality. The use of Daoyin has no adverse effects and side effects and can reduce the cost of medication. Therefore, Daoyin can be widely promoted. Further research is warranted to analyze the effect of Daoyin on mental health.

**Systematic review registration:**

https://www.crd.york.ac.uk/PROSPERO/#recordDetails, identifier CRD42023391845.

## 1 Introduction

COVID-19 has caused global crises in social, economic, and health sectors. Simultaneously, to a considerable degree, it has caused adverse impacts on individuals’ psychological well-being. During the COVID-19 treatment and rehabilitation periods, the mental health status of patients needs to be closely watched. Many COVID-19 patients have anxiety, depression, and sleep disorders, which have a serious impact on their physical and mental health (1–3). The COVID-19 treatment process has been significantly influenced by Traditional Chinese Medicine (TCM). To better help COVID-19 patients recover their physical and mental health, a variety of TCM Daoyin has been used in clinical adjuvant treatment. Daoyin is a form of health maintenance that involves a combination of breathing and limb movements. This practice was known to significantly improve the physical and mental well-being of individuals. Commonly used Daoyin techniques include Baduanjin, Yijinjing, Wuqinxi, and Tai Chi. In recent years, research on Daoyin has mostly focused on mental health problems, such as anxiety, depression, fatigue, and stress (4–6).

Multiple guidelines (7–10) have indicated that the use of TCM Daoyin has significantly contributed to the improvement in psychological recovery among individuals affected by COVID-19. Numerous studies have documented the use of Baduanjin in addressing anxiety, depression, insomnia, lung function recovery, immune system improvement, and physical endurance associated with COVID-19. Through the combination of exercise and breathing, it can effectively relieve anxiety and depression and improve sleep quality and body immunity. At the same time, TCM Daoyin can also promote the flow of energy inside the body, improve the circulation of Qi and blood, relieve fatigue and pressure, and improve the body’s self-healing ability. In summary, TCM Daoyin plays a crucial role in the management of individuals with COVID-19, effectively improving mental and physical recovery. The objective of our research is to thoroughly examine the adjuvant therapeutic impact of Daoyin in individuals suffering from COVID-19. Additionally, we aim to investigate the efficacy and practicality of Daoyin in clinical practice, and furnish more advanced clinical proof for the application of Daoyin.

## 2 Methods

The study protocol was registered on the International Prospective Register of Systematic Reviews (PROSPERO) (CRD42023391845). The study followed the referred Reporting Items for Systematic reviews and Meta-Analyses (PRISMA) checklist (11) and Cochrane Handbook (12) guidelines for conducting systematic reviews and meta-analyses.

### 2.1 Data source and search strategy

We conducted a comprehensive search of several databases, including PubMed, the Cochrane library, Embase, CNKI, Wanfang, VIP database, and SinoMed from their establishment until February 2024. MeSH words and free words or phrases and their abbreviations were used as a search strategy and modified to fit the style of each Chinese and English database. The search terms consisted of two parts (1): COVID-19 (MeSH); 2019-nCoV Infection; SARS-CoV-2 Infection; 2019 Novel Coronavirus Disease; COVID-19 Virus Infection; Coronavirus Disease 2019; (2) Daoyin (MeSH); Traditional Chinese Exercise; Qigong; Gongfa; Baduanjin; BDJ; Yijinjing; YJJ; Wuqinxi; WQX; Tai Ji Quan; TJQ; Taichi; Liuzijue; pulmonary rehabilitation. Additionally, we manually searched for studies from alternative sources that satisfied the inclusion and exclusion criteria but were not included in the aforementioned databases. Two researchers (N.F. Duan and F. Chen) independently selected the appropriate studies. All databases were searched in any language.

### 2.2 Inclusion and exclusion criteria

#### 2.2.1 Participants

This study exclusively involved individuals diagnosed with COVID-19.

#### 2.2.2 Interventions

Included in the studies were comparisons between Daoyin plus usual care and usual care, regardless of whether blinding was implemented. The Daoyin methods include but are not limited to Baduanjin, Yijinjing, Wuqinxi, Tai Ji Quan, Liuzijue, pulmonary rehabilitation, etc. The defined exclusion criteria consisted of (1) excluding case reports, case series, reviews, editorials, and animal studies; (2) excluding scale results that are not presented as scores; (3) excluding results that do not meet the statistical data format requirements; (4) excluding duplicate publications that report the same results.

#### 2.2.3 Controls

The control interventions solely consisted of usual care. Usual care primarily involves pharmacotherapy, encompassing TCM and antiviral medications, among other interventions (13).

#### 2.2.4 Outcomes

We assessed the main measures of outcomes, which encompassed (1) scores from the Self-Rating Anxiety Scale (SAS) and (2) scores from the Self-Rating Depression Scale (SDS). The secondary outcome measures we evaluated included: (1) Pittsburgh Sleep Quality Index scores (PSQI scores); (2) Hamilton Anxiety Scale scores (HAMA scores); and (3) Hamilton Depression Scale scores (HAMD scores).

#### 2.2.5 Study design

Only Randomized Controlled Trials (RCTs) was considered for inclusion. These studies focused on examining the effectiveness and safety of Daoyin in treating COVID-19 patients with psychological symptoms.

### 2.3 Data collection, extraction, and evaluation

#### 2.3.1 Study selection

Two researchers (N.F. Duan and F. Chen) individually reviewed the titles and abstracts of the studies that were obtained, assessing them based on predetermined criteria and documenting their judgments accordingly. If a disagreement occurred, it was discussed with another author (W. Zhang).

#### 2.3.2 Data extraction

Two independent researchers (N.F. Duan and F. Chen) examined all the research papers and collected pertinent information based on pre-established criteria. The primary information obtained from the research encompassed the names of the authors, year of publication, geographical location of publication, study design type, method of randomization, case count, age range, method of blinding, criteria for inclusion and exclusion, diagnostic criteria, criteria for evaluating efficacy, methods of intervention, measures of outcome, and duration of treatment. If missing data were found, we contacted the original corresponding author via email to obtain sufficient information. Only one study was included when multiple studies reported the identical trial. If any missing data were discovered, the authors in charge of the correspondence were reached out to via email to provide the necessary information.

#### 2.3.3 Evaluation of the risk of bias

The assessment of bias was conducted based on the following criteria: (1) random sequence generation (selection bias); (2) allocation concealment (selection bias); (3) blinding of participants and personnel (performance bias); (4) blinding of outcome assessment (detection bias); (5) incomplete outcome data (attrition bias); (6) selective outcome reporting (reporting bias); and (7) other biases. Every possible factor that could introduce bias was assessed and categorized as either high, low, or uncertain. High risk is indicated by the color red, unclear risk is indicated by the color yellow, and low risk is indicated by the color green. Data were extracted and bias risk was evaluated by two researchers (N.F. Duan and F. Chen) individually, using the criteria table from the Cochrane Handbook of 2019. If a disagreement occurred, it was discussed with another author (W. Zhang).

### 2.4 GRADE evaluation

To evaluate the quality of the evidence, the Grading of Recommendations, Assessment, Development and Evaluation (GRADE) system was employed (14, 15). The initial score for the quality of evidence for the RCT was 4 and then downgraded according to the five factors of risk of bias, inconsistency, indirectness, imprecision, and publication bias. The evidence was categorized into four levels of certainty: ‘high’, ‘moderate’, ‘low’, and ‘very low’. Two researchers (Y.L.Li and L.Q.Zhou) conducted the GRADE evaluation on their own and any differences were resolved by a third researcher (G.H.Xu).

### 2.5 Data synthesis and statistical analysis

The analysis and presentation of the findings were conducted using Review Manager 5.2 software. Continuous variables were determined using mean difference (MD), inverse variance, and 95% confidence intervals (CIs). The range of *I^2^* values was between 0% and 100%, and they were classified as follows: *I^2^* ollows which may not be significant; 30% < *I^2^* 0% < i indicating moderate heterogeneity; 50% < *I^2^* 0% < g suggesting substantial heterogeneity; and 75% < *I^2^* 5% < %, indicating considerable heterogeneity (16). The estimates were combined using a fixed-effect model. Subgroup and sensitivity analyses were used to identify potential sources of heterogeneity. Statistically significant differences were deemed at *P*-values below 0.05. Subgroup analysis was performed on each Daoyin method. Forest plots are used to present the findings. Publication bias was analyzed using funnel plot and Egger’s analysis.

## 3 Results

### 3.1 Literature selection

**Figure 1:**
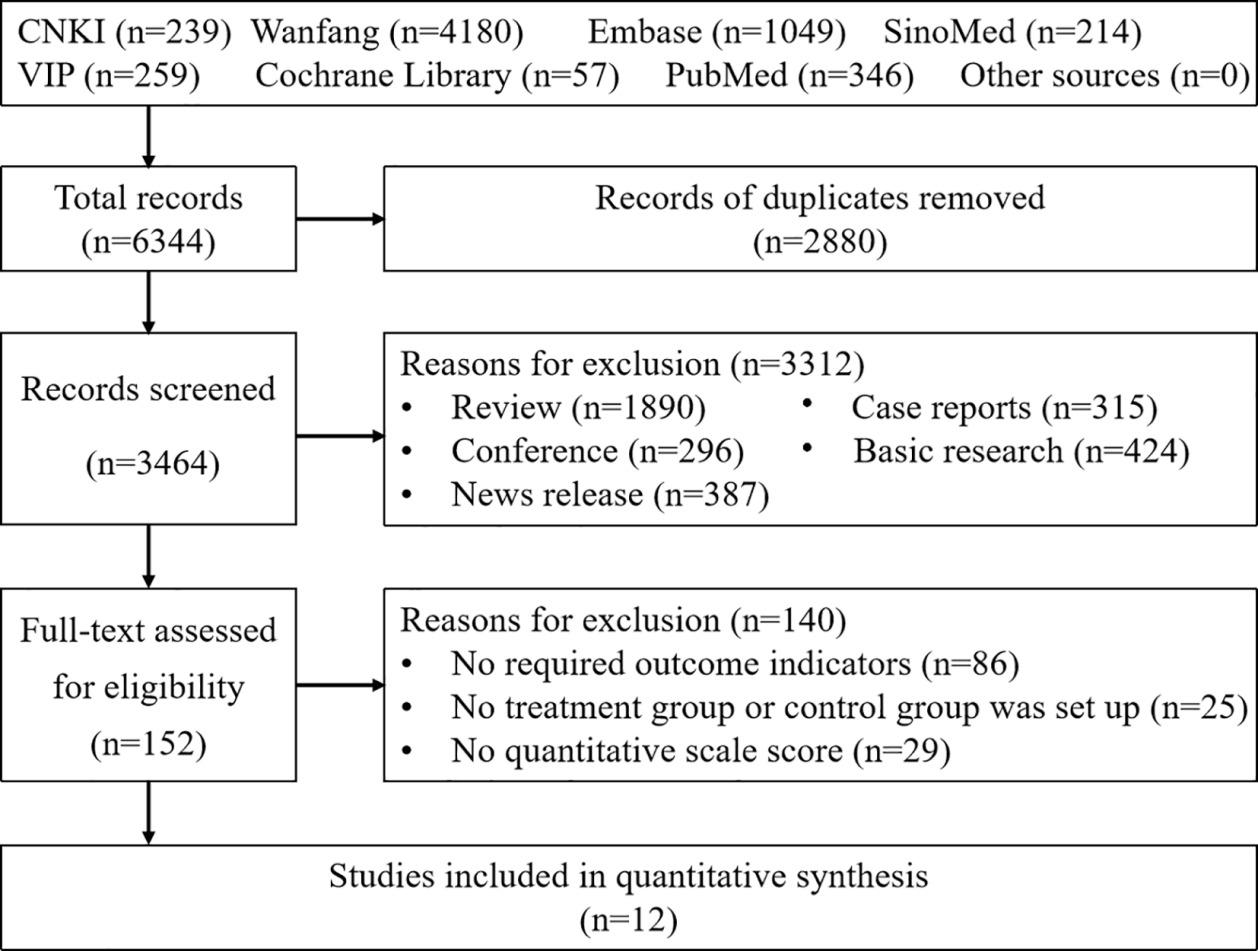
The PRISMA flowchart of the study search and selection process.

A total of 6,344 articles were retrieved by searching CNKI (239), Wanfang (4180), VIP (259), SinoMed (214), PubMed (346), Embase (1049) and the Cochrane library (57). A total of 2,880 articles were excluded by duplicate checking. After reading the abstract and full text, 3,312 and 140 articles were excluded, respectively. Ultimately, 12 RCTs involving 1,291 cases were included (17–28). The screening process is presented in **Figure 1**.

### 3.2 Characteristics of the included studies

There were 651 and 640 cases in the treatment and control groups, respectively. All the studies were published in China, including four Daoyin methods: Baduanjin, Yijinjing, Liuzijue, and Kangyi Qiangshen Gong. Publication years were between 2020 and 2022. The studies exhibited consistent baseline characteristics. Two researchers independently performed data extraction on the included studies. **Table 1** provides a comprehensive overview of the specific attributes of the research.

**Table 1:** Characteristics of the included studies.

Study Year Region	Type	Daoyin methods	SARS-CoV-2	Cases (treatment group/control group)	Age (Treatment group/Control group)	Intervention		Course of treatment	Outcomes
						Treatment group	Control group		
Chen 2020 (17) China	RCT	Baduanjin	+	14/15	41−82 (67.6 ± 11.2)/ 39−84 (68.5 ± 10.8)	Baduanjin+usual care, 20–3 0min/time, 2 times/d	Usual care	21	1, 2
Wu 2020 (18) China	RCT	Baduanjin	+	60/60	51.3 ± 8.45/51.3 ± 8.45	Baduanjin+usual care, 30–60 min/time, 2 times/d	Usual care	15	1
Yang 2020 (19) China	RCT	Baduanjin	+	55/55	48−76 (58.69 ± 5.41)/ 47−75 (58.23 ± 5.36)	Baduanjin+acupressure (Tiantu, Danzhong, Dazhui)+usual care, 30 min/time, 1 time/d	Usual care	30	1, 2
Zhang 2020 (20) China	RCT	Yijinjing	+	14/14	39−60 (49.87 ± 4.23)/ 39−62 (49.89 ± 4.22)	Yijinjing+usual care, 60 min/time, 2 times/d, 3 d/week	Usual care	7	1, 2
Li 2021 (21) China	RCT	Liuzijue	+	30/30	19−67 (46.33 ± 13.76)/ 18−65 (43.2 ± 12.94)	Liuzijue+five Elements music+usual care, 30 min/time, 2 times/d	Usual care	14	1, 3, 4
LiZ 2021 (22) China	RCT	Baduanjin	+	59/44	55.7 ± 13.5/ 56.9 ± 13.7	Baduanjin+usual care, 30–60 min/time, 2 times/d, 5 d/week	Usual care	14	4
Wang 2021 (23) China	RCT	Baduanjin	+	30/30	19−75 (63.41 ± 6.73)/ 18−73 (63.42 ± 6.91)	Baduanjin+five Elements music+usual care, 30 min/time, 2 times/d	Usual care	14	4, 5
Yang 2021 (24) China	RCT	Baduanjin	+	42/45	21−65 (45 ± 11)/ 22−65 (45 ± 11)	Baduanjin+usual care, 30 min/time, 1 times/d	Estazolam Tablets+usual care, 1 mg/time, 1 times/d	12	1, 2, 3
Yin 2021 (25) China	RCT	Baduanjin	+	20/20	29−63 (47.1 ± 10.99)/ 20−66 (43.95 ± 13.75)	Baduanjin+five Elements music+usual care, 60 min/time, 1 times/d, 5 d/week	Usual care	14	1, 2
Cai 2022 (26) China	RCT	Yijinjing	+	30/30	43.5 ± 17.5/ 42.4 ± 17.8	Yijinjing+Usual care, 30 min/time, 1 times/d	Usual care	14	3, 5
Wen 2022 (27) China	RCT	Kangyi Qiangshen Gong	+	290/290	52.94 ± 15.76/ 49.52 ± 16.28	Kangyi Qiangshen Gong+Usual care, 20 min/time, 1 times/d	Usual care	7	1
Yang 2022 (28) China	RCT	Baduanjin	+	7/7	1−60 (35.36 ± 8.22)/ 27−69 (49.88 ± 7.13)	Baduanjin+Usual care, 12 min/time, 2 times/d	Usual care	8−12	1, 2

The “+”means positive for severe acute respiratory syndrome coronavirus 2 (SARS-CoV-2). RCT, Randomized Controlled Trial. The outcomes are the Self-Rating Anxiety Scale (SAS), Self-Rating Depression Scale (SDS), Pittsburgh Sleep Quality Index (PSQI), Hamilton Anxiety Scale (HAMA), and Hamilton Depression Scale (HAMD), which are denoted by “1”, “2”, “3”, “4”, and “5”, respectively. The usual care primarily involves pharmacotherapy, encompassing TCM and antiviral medications, among other interventions.

### 3.3 Risk of bias assessment

**Figure 2:**
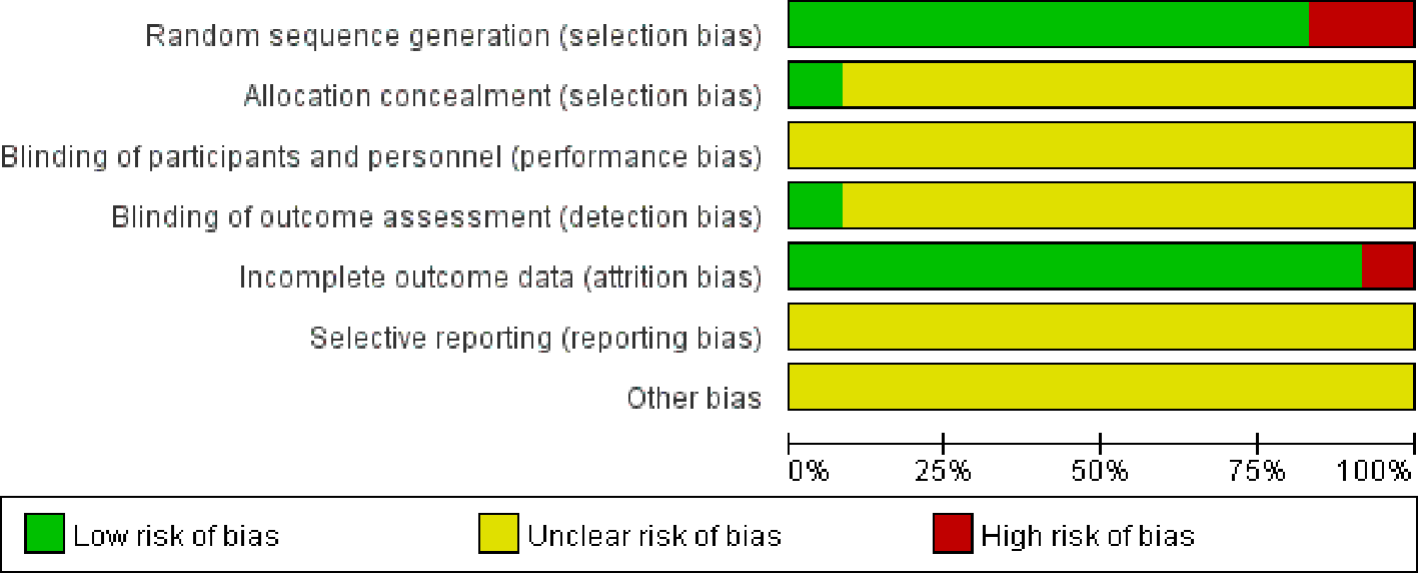
Assessment of risk of bias in the included studies.

**Figure 3:**
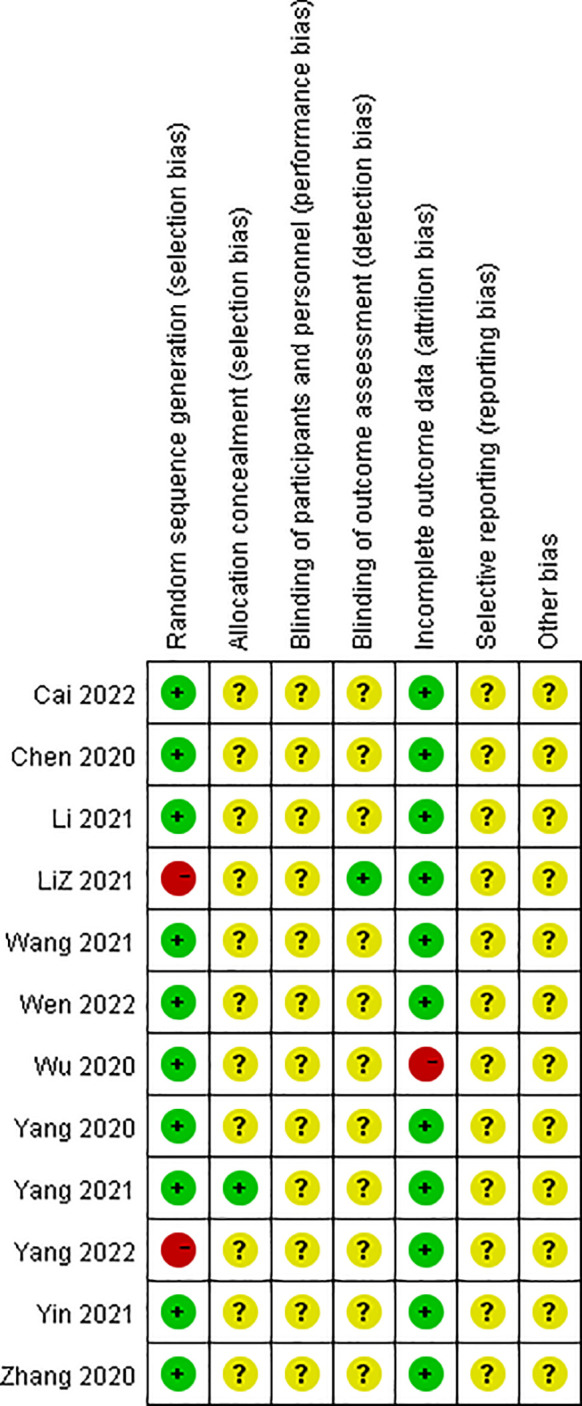
Assessment of the risk of bias summary in the included studies.

The risk bias assessment details for the 12 studies included are depicted in **Figures 2** and **3**. In the context of random sequence generation, 10 studies employed the randomization method and were assessed as having a low risk of bias, nine of which (17–21, 23, 25–27) used the random number table method and one (24) obtained a random number through a central randomization system. Of the remaining 2 studies, one study did not mention the randomization method (22) and the other (28) was grouped by patient age and gender, which was rated as high risk. In the section pertaining to allocation concealment, one study (24) provided a detailed description of the allocation concealment method, resulting in a low risk rating. A separate study (22) implemented blinding during outcome assessment and was deemed to have a low risk of bias. In the data integrity section, one study (18) reported case shedding without reason and was rated as high risk. For selective reporting and other bias sections, all studies did not mention this section and were rated as unclear risk.

### 3.4 Primary outcome: SAS

**Figure 4:**
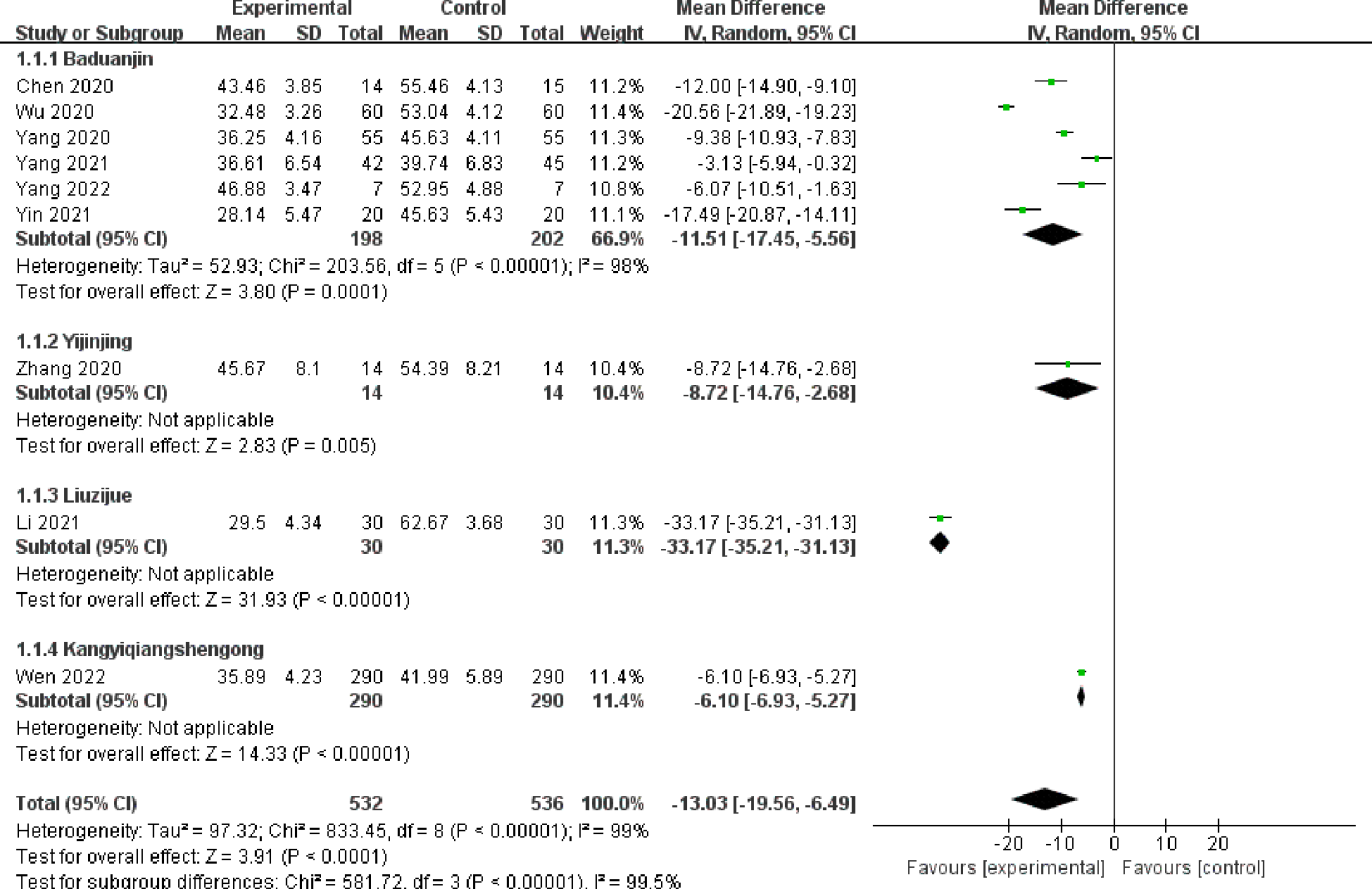
Forest plot of SAS scores.

Nine studies (17–21, 24, 25, 27, 28) comprising 1,068 patients evaluated the differences in SAS scores between the Daoyin treatment (532 patients) group and control (536 patients) group (**Figure 4**). Nine studies including four Daoyin methods: Baduanjin, Yijinjing, Liuzijue, and Kangyi Qiangshen Gong. Among them, six studies used the Baduanjin method. The findings indicated that the integrated Daoyin intervention led to a statistically significant decrease in the SAS score (MD = −13.03, 95% CI: −19.56 to −6.49, *P <*0.0001). The findings of the heterogeneity test indicated a significantly high level of statistical heterogeneity among the studies (*I^2^* = 99%, *P <*0.00001), leading to the use of the random effects model for data analysis.

### 3.5 Primary outcome: SDS

**Figure 5:**
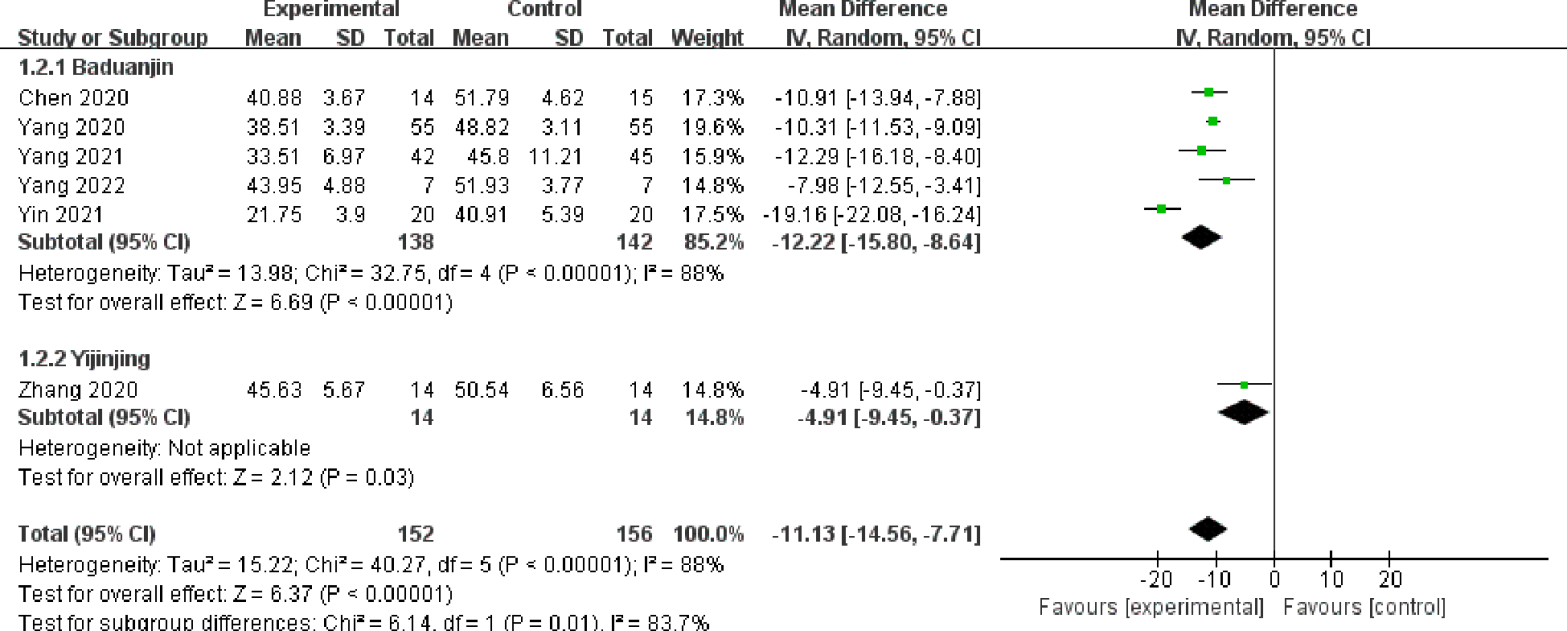
Forest plot of SDS scores.

Six studies (17, 19, 20, 24, 25, 28) involving 308 cases reported the changes in SDS scores (**Figure 5**). There were 152 patients and 156 patients in the treatment and control groups, respectively. Six studies included two Daoyin methods: Baduanjin and Yijinjing. Among them, five studies used the Baduanjin method. According to the findings, the group receiving Daoyin treatment exhibited a significant decrease in SDS scores compared with the control group (MD = −11.13, 95% CI: −14.56 to −7.71, *P <*0.00001). There was a significant level of heterogeneity (*I^2^* = 88%, *P* < 0.00001). The data were analyzed using a random effects model.

### 3.6 Secondary outcome: PSQI

**Figure 6:**
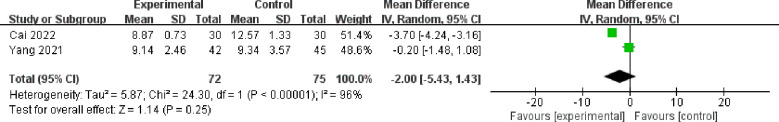
Forest plot of PSQI scores.

Changes in the PSQI scores were assessed in two studies (24, 26) including 147 patients (**Figure 6**). There were 72 patients and 75 patients in the treatment and control groups, respectively. The results showed that the combined Daoyin intervention could reduce the PSQI scores (MD = −2.00, 95% CI: -−5.43 to 1.43, *P* = 0.25). Although the results were not statistically significant, there was a trend toward lower PSQI scores. The degree of heterogeneity was found to be substantial (*I^2^* = 96%, *P* < 0.00001), leading to the use of the random effects model for data analysis.

### 3.7 Secondary outcome: HAMA

**Figure 7:**
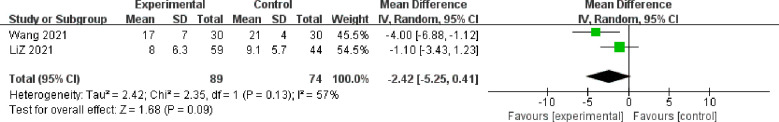
Forest plot of HAMA scores.

Two studies (22, 23) involving 163 cases reported the changes in HAMA scores (**Figure 7**). There were 89 patients and 74 patients in the treatment and control groups, respectively. The findings indicated that the Daoyin treatment group exhibited a statistically significant reduction in HAMA scores compared with the control group (MD = −2.42, 95%CI: −5.25 to 0.41, *P* = 0.09). Although the results were not statistically significant, there was a trend toward lower HAMA scores. High heterogeneity was present (*I^2^* = 57%, *P* = 0.13); therefore, a random effects model was used for data analysis.

### 3.8 Secondary outcome: HAMD

**Figure 8:**
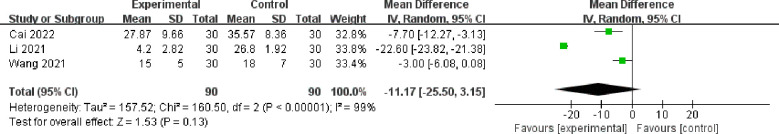
Forest plot of HAMD scores.

Changes in the HAMD scores were assessed in three studies (21, 23, 26) including 180 patients (**Figure 8**). There were 90 patients each in the treatment and control groups. The results showed that the combined Daoyin intervention could reduce the HAMD scores (MD = −11.17, 95%CI: −25.5 to 3.15, *P* = 0.13). Although the results were not statistically significant, there was a trend toward lower HAMD scores. There was a significant high level of heterogeneity (*I^2^* = 99%, *P* < 0.00001). The data were analyzed using the random effects model.

### 3.9 Safety analysis: adverse effects

All studies had no adverse effects during the whole research process.

### 3.10 Subgroup analysis

Subgroup analysis was performed using different Daoyin methods as classification. For the SAS scores, 6 (17–19, 24, 25, 28) out of 9 studies used Baduanjin. The subgroup analysis results indicated significant heterogeneity among the studies (*I^2^* = 98%, *P <*0.00001). The remaining 3 studies (20, 21, 27) used the Daoyin methods of Yijinjing, Liuzijue, and Kangyi Qiangshen Gong. Heterogenetic analysis could not be performed due to a single study. There were no subgroup heterogeneous data in these three studies. In term of SDS scores, 5 (17, 19, 24, 25, 28) of 6 studies used the Baduanjin Daoyin method, and heterogeneity was still high (*I^2^* = 88%, *P <*0.00001).

### 3.11 Sensitivity analysis

Sensitivity analysis was conducted to assess potential sources of heterogeneity through systematic removal of individual studies to observe changes in the data. For the SAS scores, the heterogeneity data remained 99% or 98% after removing the studies individually. There was no significant change observed in the heterogeneity results. For the SDS scores, most heterogeneity data were approximately 90%. When only one study was removed, the heterogeneity data decreased to 46% (details are shown in **Table 2**).

**Table 2:** Mean difference (MD) and heterogeneity tests of outcomes for sensitivity analyses.

Excluded study	SAS		SDS	
	Pooled MD (95% CI)	P heterogeneity;I^2^	Pooled MD (95% CI)	P heterogeneity;I^2^
No studies were removed	−13.03 (−19.56, −6.49)	<0.00001; 99%	−11.13 (−14.56, −7.71)	<0.00001; 88%
Chen 2020 (14)	−13.15 (−20.32, −5.98)	<0.00001; 99%	−11.13 (−15.43, −6.82)	<0.00001; 90%
Wu 2020 (15)	−12.06 (−19.18, −4.94)	<0.00001; 99%		
Yang 2020 (16)	−13.47 (−21.26, −5.68)	<0.00001; 99%	−11.22 (−16.12, −6.32)	<0.00001; 89%
Zhang 2020 (17)	−13.52 (−20.48, −6.57)	<0.00001; 99%	−12.22 (−15.80, −8.64)	<0.00001; 88%
Li 2021 (18)	−10.49 (−15.52, −5.46)	<0.00001; 98%		
Yang 2021 (21)	−14.27 (−21.29, −7.24)	<0.00001; 99%	−10.88 (−14.92, −6.84)	<0.00001; 90%
Yin 2021 (22)	−12.47 (−19.52, −5.41)	<0.00001; 99%	−9.78 (−11.66, −7.89)	=0.11; 46%
Wen 2022 (24)	−13.91 (−20.96, −6.87)	<0.00001; 99%		
Yang 2022 (25)	−13.87 (−20.86, −6.87)	<0.00001; 99%	−11.67 (−15.53, −7.81)	<0.00001; 90%

### 3.12 Evaluation of publication bias

**Figure 9:**
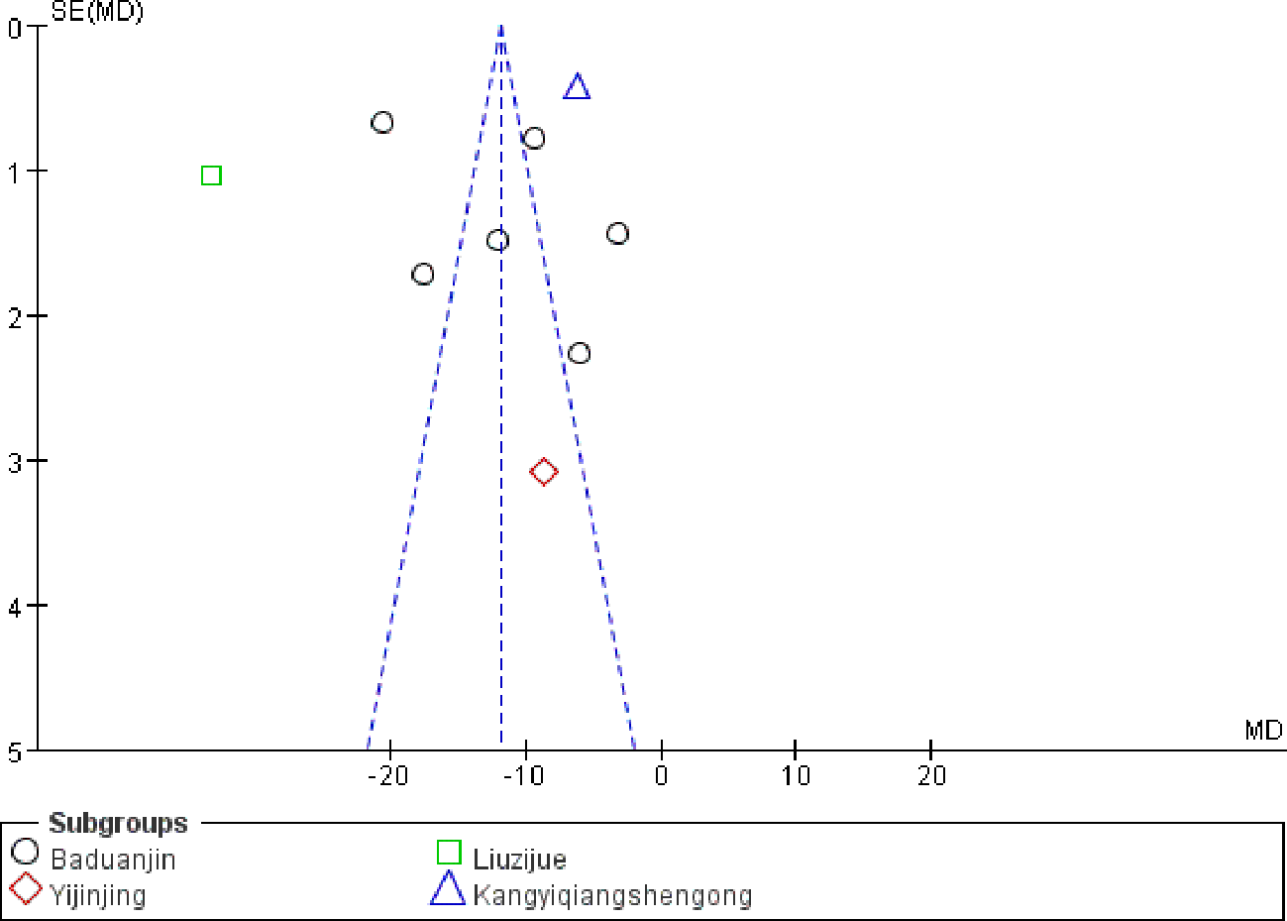
Funnel plot of SAS scores.

The SAS scores data were used to draw the funnel plot (**Figure 9**). A total of nine studies (17–21, 24, 25, 27, 28) were included. The symmetrical nature of the figure implies the absence of any discernible publication bias. An Egger’s analysis was further performed (*P* = 0.086 > 0.05), indicating the absence of significant publication bias.

### 3.13 Certainty of evidence assessed by the GRADE system

The certainty of evidence was assessed using the GRADE system, with the overall quality deemed to be of low and very low levels (**Table 3**). The reasons for the potential downgrading of the study’s quality related to the high risk of bias associated with the randomization and blinding methods. For inconsistency, the downgrading was due to all *I^2^* values being greater than 50%, and the forest plot shows a poor overlap. For indirectness, the study population, intervention methods and outcome indicators have good clinical applicability; therefore, it is not downgraded. The reasons for the downgrade were attributed to imprecision, as evidenced by the small sample size and wide range of confidence intervals in the outcome of the HAMD. The remaining outcomes were not downgraded. For publication bias, there was no publication bias such as corporate funding; therefore, it was not downgraded.

**Table 3:** The quality of outcomes evaluated using the GRADE system.

Certainty assessment							Summary of findings			
Outcomes (Number of studies)	Study design	Risk of bias	Inconsistency	Indirectness	Imprecision	Publication bias	Number of patients		Effect	Certainty
							Treatment group	Control group	MD (95% CI)	
SAS (9)	RCT	Serious^a^	Serious^b^	Not serious	Not serious	Not serious	532	536	−13.03 [−19.56, −6.49]	⨁⨁◯◯ Low
SDS (6)	RCT	Serious^a^	Serious^b^	Not serious	Not serious	Not serious	152	156	−11.13 [−14.56, −7.71]	⨁⨁◯◯ Low
PSQI (2)	RCT	Serious^a^	Serious^b^	Not serious	Not serious	Not serious	72	75	−2.00 [−5.43, 1.43]	⨁⨁◯◯ Low
HAMA (2)	RCT	Serious^a^	Serious^b^	Not serious	Not serious	Not serious	89	74	−2.42 [−5.25, 0.41]	⨁⨁◯◯ Low
HAMD (3)	RCT	Serious^a^	Serious^b^	Not serious	Serious^c^	Not serious	90	90	−11.17 [−25.5, 3.15]	⨁◯◯◯ Very low

The outcomes are Self-Rating Anxiety Scale (SAS), Self-Rating Depression Scale (SDS), Pittsburgh Sleep Quality Index (PSQI), Hamilton Anxiety Scale (HAMA) and Hamilton Depression Scale (HAMD). RCT, Randomized Controlled Trial; MD, Mean Difference. a, randomization and blinding had a high risk and unclear risk of bias; b, heterogeneity I^2^ was greater than 50% and had poor inter-study coincidence; c, wide credible interval and a small sample size.

## 4 Discussion

In the present meta-analysis, 12 RCTs involving 1,291 cases were included involving four Daoyin methods: Baduanjin, Yijinjing, Liuzijue, and Kangyi Qiangshen Gong. The results showed a significant reduction in SAS and SDS scores. Although the PSQI, HAMA, and HAMD scores did not demonstrate statistical significance, they did exhibit a significant decreasing trend. The aforementioned findings suggest that Daoyin has a significant impact on the regulation of the mental health and sleep of individuals affected by COVID-19. However, the heterogeneity of the included studies was relatively high. As different Daoyin methods have their own characteristics, this may lead to higher clinical heterogeneity. Therefore, we performed subgroup analysis using different Daoyin methods as groups. The results showed that the subgroup analysis did not significantly reduce the heterogeneity, which may be due to the uneven distribution of the number of cases and the large difference in the improvement of outcome indicators. Following that, sensitivity analysis was conducted and revealed no substantial alteration in the heterogeneity findings, suggesting that the incorporated studies remained relatively stable. We used two methods, funnel plotting and Egger’s analysis, to verify that there was no publication bias in this study. The certainty of evidence was evaluated using the GRADE system, and the overall quality was determined to be low and very low. By integrating these data, the study demonstrated that combined Daoyin treatment can significantly improve anxiety, depression, and sleep status in COVID-19 patients. As a result of the disparate levels of evidence present in the studies that were incorporated, a higher level of evidence is still needed to verify this result.

COVID-19 represents a significant public health crisis characterized by its extensive scope and profound implications. It has a great psychological impact on the patients and can easily cause negative emotions, such as panic and anxiety. Therefore, it is of great significance to explore targeted psychological crisis intervention programs to relieve negative emotions, reduce psychological stress, and maintain a stable attitude. Studies have shown that the literature on the behavioral and psychosocial aspects of the COVID-19 pandemic has been dominated by topics about stress or distress (29). A significant proportion of individuals who had recuperated from acute COVID-19 infection experienced persistent symptoms that significantly impacted their daily functioning and quality of life. These individuals are known as long COVID-19 patients. Even after recovering from the infection, individuals may experience lingering atypical chronic symptoms, such as profound tiredness, breathing difficulty, joint discomfort, cognitive impairment, uneasiness, and sadness for several months, indicating that the fundamental disease pathology continues to exist (30). According to a meta-analysis (31) of 151 studies with 1,285,407 individuals from 32 different nations, a minimum of 50.1% of individuals who recovered from COVID-19 experienced long-term effects within a year following the infection. The most frequent abnormalities were pulmonary CT (56.9%) and lung function tests (45.6%), followed by fatigue (28.7%), depression (18.3%), and post-traumatic stress disorder (17.9%). Based on another meta-analysis (32), fatigue (27.4%) was found to be the most prevalent consequence within 2 years of contracting COVID-19, closely followed by sleep problems (25.1%). The main focus now is to offer suitable measures for prevention and intervention management to reduce the long-term consequences and improve the overall well-being of individuals who have recovered from COVID-19. Our study used the SAS and SDS as the main study indicators. These two scales are used to assess how well individuals feel in terms of anxiety and depression and help people better understand their mental health status. Our study findings indicated that the inclusion of Daoyin therapy can effectively reduce the levels of anxiety and depression experienced by patients. Within the current social background, it can provide a variety of options for the treatment of psychological problems.

A total of 4 Daoyin methods were included in our study, including Baduanjin, Yijinjing, Liuzijue, and Kangyi Qiangshen Gong. The shared characteristic among these Daoyin methods is the integration of respiratory and limb movements, with the goal of preventing and treating illnesses by altering body posture, regulating breathing, and modifying psychological states. Baduanjin comprises eight graceful movements that are relatively uncomplicated and easily mastered, making it a widely accepted instructional method. This exercise routine seamlessly blends soft and firm elements, incorporating both dynamic and static components. Practitioners are encouraged to synchronize their breathing, physical movements, and mental focus to achieve a state of relaxation during physical and psychological workouts (33). One study (34) showed that Baduanjin, as an auxiliary rehabilitation method, can improve psychological and physiological parameters in different age groups and different clinical populations, and effectively improve cognitive function. According to an RCT (35), Baduanjin can decrease depression in individuals without any health issues and increase oxygenated hemoglobin levels in the left prefrontal cortex of the brain. The results suggest that Baduanjin is a beneficial workout for improving executive function and potentially regulating brain activity in individuals who are in good health. One study (36) explored the mechanism of Baduanjin and showed that it alleviates diabetes and depression by regulating the expression of mRNA, lncRNA, and circRNA. KEGG and GO enrichment analysis showed that the pathway mainly focused on immune function and inflammatory response, including the IL-17 signaling pathway and TNF signaling pathway. No further analysis was carried out in the study. Although the mechanism was not fully explored, it provides guidance for the further development of Baduanjin. The aforementioned research indicates that Baduanjin plays a crucial role in the regulation of mental well-being for individuals who are both in good health and those who are unwell. Yijinjing, an ancient Chinese medical practice, encompasses 12 movements and is regarded as a form of complementary and alternative medicine used for maintaining health, providing medical care, and treating diseases. Yijinjing employs a variety of pulling and stretching exercises to promote relaxation of the body’s muscles and joints, ultimately aiming to improve blood circulation, alleviate pain, and reduce fatigue (37). The practice of Yijinjing has been shown to have a positive impact on sleep disorders, fatigue, and overall quality of life in individuals with chronic fatigue syndrome. Additionally, it has been found to be more effective than behavioral cognitive education in reducing pain and increasing vitality (38). The essence of Liuzijue lies in the regulation of exhalation and inspiration through the production of six distinct sounds. The pronunciation of Xu, He, Hu, Si, Chui, and Xi, six words of different mouth, lip, teeth, and tongue force, affect different viscera meridians qi and blood operations (39). Liuzijue is a promising rehabilitation exercise program for discharged COVID-19 patients. Four weeks after the intervention, the patient’s lung function was improved, dyspnea was relieved, and exercise capacity was improved. It can also significantly relieve the state of depression and anxiety (40). Kangyi Qiangshen Gong is a combination of various traditional Daoyin methods, such as Taijiquan, Baduanjin, Yijinjing, and Liuzijue (41). The researchers selected targeted movements from various Daoyin methods and optimized them for patients with clinical symptoms such as dyspnea, chest tightness, fatigue, and cough. Kangyi Qiangshen Gong can improve lung function and immunity and is widely used in the rehabilitation and adjuvant treatment of lung-related diseases (42).

The above studies expressed the significant improvement effect of Daoyin on COVID-19, respiratory diseases, and psychological diseases, and contributed more options for clinical treatment. In comparison with pharmaceutical interventions, Daoyin exercises can decrease healthcare expenditures and alleviate the physical and financial strain on individuals, which deserves wider promotion.

## 5 Conclusion

To summarize, the existing evidence indicates that Daoyin has a notable impact on alleviating anxiety, depression, and sleep disturbances in individuals suffering from COVID-19. Nevertheless, as a result of the inadequate standard of the studies incorporated in this examination and the findings indicating substantial heterogeneity, there is a need to increase the level of evidence. In the future, it is necessary to conduct additional validation of high-quality well-designed multi-center RCTs.

## Data Availability

The original contributions presented in the study are included in the article/**Supplementary Material**. Further inquiries can be directed to the corresponding author.
